# Mesenchymal Stem Cell Therapy for Ischemic Tissues

**DOI:** 10.1155/2018/8179075

**Published:** 2018-10-08

**Authors:** Kar Wey Yong, Jane Ru Choi, Mehdi Mohammadi, Alim P. Mitha, Amir Sanati-Nezhad, Arindom Sen

**Affiliations:** ^1^Pharmaceutical Production Research Facility, Department of Chemical and Petroleum Engineering, Schulich School of Engineering, University of Calgary, 2500 University Drive NW, Calgary, AB, T2N 1N4, Canada; ^2^BioMEMS and Bioinspired Microfluidic Laboratory, Department of Mechanical and Manufacturing Engineering, Schulich School of Engineering, University of Calgary, 2500 University Drive NW, Calgary, AB, T2N 1N4, Canada; ^3^Department of Mechanical Engineering, University of British Columbia, 2054-6250 Applied Science Lane, Vancouver, BC, V6T 1Z4, Canada; ^4^Centre for Blood Research, Life Sciences Centre, University of British Columbia, 2350 Health Sciences Mall, Vancouver, BC, V6T 1Z3, Canada; ^5^Department of Biological Sciences, University of Calgary, 2500 University Drive NW, Calgary, AB, T2N 1N4, Canada; ^6^Department of Clinical Neurosciences, Foothills Medical Centre, University of Calgary, 3330 Hospital Drive NW, Calgary, AB, T2N 4N1, Canada; ^7^Center of Bioengineering Research and Education, Schulich School of Engineering, University of Calgary, 2500 University Drive NW, Calgary, AB, T2N 1N4, Canada

## Abstract

Ischemic diseases such as myocardial infarction, ischemic stroke, and critical limb ischemia are immense public health challenges. Current pharmacotherapy and surgical approaches are insufficient to completely heal ischemic diseases and are associated with a considerable risk of adverse effects. Alternatively, human mesenchymal stem cells (hMSCs) have been shown to exhibit immunomodulation, angiogenesis, and paracrine secretion of bioactive factors that can attenuate inflammation and promote tissue regeneration, making them a promising cell source for ischemic disease therapy. This review summarizes the pathogenesis of ischemic diseases, discusses the potential therapeutic effects and mechanisms of hMSCs for these diseases, and provides an overview of challenges of using hMSCs clinically for treating ischemic diseases.

## 1. Introduction

Ischemic diseases are usually characterized as a reduced blood flow to a tissue or organ due to undesirable vascular conditions, such as blood vessel stenosis or aneurysm rupture [1]. Myocardial infarction, ischemic stroke, and critical limb ischemia are the three most common ischemic diseases [2]. Reperfusion therapy (pharmaceutical or surgical approach) is typically used to counteract these diseases. The clinical administration of pharmaceuticals, such as thrombolytic agents (e.g., recombinant tissue plasminogen activator or streptokinase) and anti-inflammatory agents (e.g., statins), is a common approach to treat the symptoms of these diseases [3, 4]. However, the systemic administration of such agents can cause a host of undesirable side effects [5]. Surgical interventions are also commonly used (e.g., stent placement to block stenotic arteries) [6]. Surgical approaches also suffer from several disadvantages: surgery always has an associated risk, disease sites may be difficult to manually access, and certain conditions are prone to recurrence (e.g., restenosis of vessels). Thereby, surgical approaches need long-term mentoring and repeated surgical procedures [7]. Although both the pharmacotherapy and surgical approaches may restore the functions of arteries, they cannot promote regeneration and functional recovery of the surrounding tissues affected by ischemia. Thus, alternative approaches are required.

**Figure 1:**
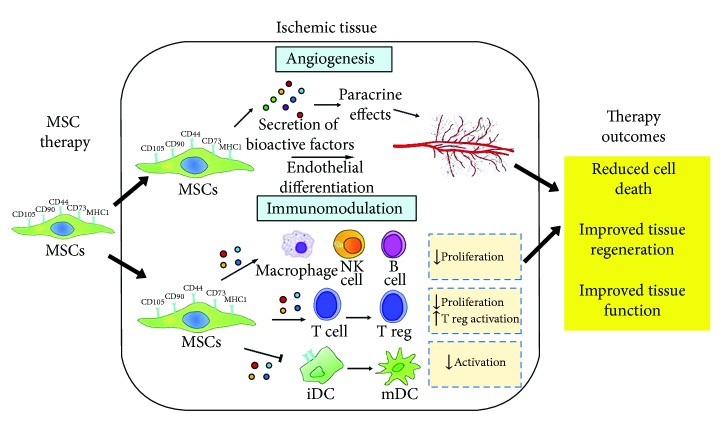
Main mechanisms of human mesenchymal stem cells (hMSCs) in the treatment of ischemic tissue. hMSCs repair ischemic tissues and restore the tissue function via angiogenesis and immunomodulation. NK: natural killer; reg: regulatory; iDC: immature dendritic cell; mDC: mature dendritic cell. This image is adapted from [12] published under the Creative Common Attribution License.

Human mesenchymal stem cells (hMSCs) can be isolated from various locations of the human body, e.g., bone marrow, adipose, and umbilical cord [8, 9]. They are capable of secreting bioactive factors for immunomodulation and angiogenesis, which can help to promote tissue repair and regeneration [10–12]. It has been shown that hMSCs may suppress the activation and functions of leukocytes actively involved in atherosclerosis, indicating their great potential in repairing injured blood vessels for the prevention of tissue ischemia [13]. If the injured blood vessel is beyond repair, hMSCs can secrete angiogenic factors (especially vascular endothelial growth factor (VEGF)) and differentiate into endothelial cells for inducing angiogenesis in ischemic regions and promote regeneration and functional recovery of injured tissues [10, 14]. In addition, protocols to expand hMSCs in culture to clinically significant levels have been reported in both the presence and absence of animal serum [15, 16]. With such fascinating properties, hMSCs can be potentially used for clinical applications in vessel repair and ischemic diseases and may be able to successfully treat ischemic tissues (Figure 1). To date, positive outcomes have been demonstrated for the treatment utility of hMSCs in preclinical trials using animal models of ischemic diseases [17–19]. Although preclinical trials have contributed much to our understanding of the pathophysiological and therapeutic mechanisms of various diseases, translation of these results to clinical trials have remained controversial [20]. There remains a lack of published clinical trials revealing the therapeutic effectiveness of hMSCs in ischemic diseases, such as myocardial infarction, ischemic stroke, and critical limb ischemia, as most of the ongoing clinical trials still remain at phase 1 for safety evaluation (http://www.clinicaltrials.gov). Both evaluation of safety (phase 1) and therapeutic efficacy (phase 2) are time-consuming due to the lack of a suitable human *in vitro* ischemic disease model for assessing the safety and effectiveness of stem cell therapy from different aspects of cell dosage, cell source, and cell administration methods and timing prior to clinical trials.

There exist several review articles focused on stem cell-based therapy for stroke [21], peripheral arterial diseases [22], and cardiovascular diseases [23]. In view of the rising demand for the use of hMSCs in ischemic disease therapy, there is a strong need for a timely review on therapeutic mechanisms of hMSCs in ischemic diseases and challenges in translating hMSCs to ischemic tissue-related clinical applications. In this review, the pathogenesis of ischemic diseases is first summarized. The potential therapeutic effects and mechanisms of hMSCs in treating myocardial infarction, ischemic stroke, and critical limb ischemia are highlighted. Lastly, the challenges associated with the future translation of hMSCs to the clinical settings in ischemic diseases are briefly discussed.

## 2. Understanding Pathogenesis of Ischemic Diseases for hMSC Therapy

Most ischemic diseases are caused by atherosclerosis, which is a chronic arterial inflammatory disease resulted from many risk factors, including hypertension, hypercholesterolemia, smoking, diabetes, and aging [24, 25]. Atherosclerosis is associated with pathologic injury and dysregulation of the endothelial cells lining the luminal wall of arteries, accumulation of lipids, smooth muscle cells, leukocytes, “foam cells”, and aggregated platelets at the arterial luminal wall, resulting in plaque formation [26–28]. Both macrophages and platelets actively secrete matrix metalloproteinases (MMPs) to induce degradation of the collagenous extracellular matrix (ECM) of a blood vessel [29, 30]. To counterbalance the MMP-mediated degradation of ECM, smooth muscle cells migrate from the outer layers of the arterial wall (tunica media and adventitia) to the tunica intima to increase the collagen production rate [31]. However, it often results in undesirable remodeling as macrophages secrete cytokines (e.g., tumor necrosis factor (TNF)-*α*, interleukin (IL)-6, and IL-1*β*) to induce apoptosis of smooth muscle cells [32]. Failure of collagen production rate to offset the ECM degradation rate results in the formation of atheromatous plaques with a thin fibrous collagenous cap [33]. At this stage, transplantation of hMSCs may suppress the functions of immune cells (MMP activity and secretion of proinflammatory cytokines) and restore collagen homeostasis, suggesting that hMSCs could be explored to treat atherosclerosis for the prevention of tissue ischemia [34].

Due to high shear stress in response to hemodynamic alteration, ruptures may easily develop in atheromatous plaques, causing bleeding which results in more recruitment of platelets and triggering of a cascade of coagulation events that leads to thrombus formation [35]. This in turn can cause stenosis of arteries, resulting in the reduction of anterograde blood flow and subsequently ischemic damage to downstream tissues [35]. For example, myocardial infarction and ischemic stroke are ischemic diseases caused by the stenosis of coronary arteries and cerebral arteries, respectively, which cause high rates of morbidity and mortality in patients [36]. Owing to the ability of hMSCs to secrete angiogenin factors and undergo endothelial differentiation, hMSCs may promote angiogenesis to restore blood flow to the ischemic tissues for tissue regeneration and functional recovery [37].

## 3. Potential Therapeutic Effects and Mechanisms of hMSCs in Ischemic Diseases

### 3.1. Myocardial Infarction

Myocardial infarction occurs upon partial or complete blockage of coronary arteries in the heart, leading to ischemia and possibly death of cardiac tissues supplied by those arteries [38]. Preclinical trials of myocardial infarction induced by coronary artery occlusion in animals have shown that hMSCs can induce angiogenesis and promote regeneration and functional recovery of the ischemic heart tissues. Local transplantation of hMSCs in animal models has been effective in inducing angiogenesis by differentiating into endothelial cells to form new blood vessels at the border zone of infarcted cardiomyocytes. These cells have shown the expression of endothelial markers CD31, CD34, CD36, Egr-3, vWf, and VEGF receptor [37, 39]. The *in vivo* differentiation of hMSCs to endothelial cells is particularly efficient when they are administered as a three-dimensional spherical cell mass (3DCM) composed of cells within a substrate containing basic fibroblast growth factor (bFGF). In fact, bFGF enhances 3D clustering of hMSCs. The cell aggregates not only support survival or persistence of hMSCs in the host but also are capable of trapping hMSC-secreted VEGF for further inducing their endothelial differentiation via the Rho/myocardin-related transcription factor-A signaling pathway [37, 40]. In addition, hMSCs may augment endogenous neovascularization through paracrine secretion of soluble factors (e.g., VEGF and bFGF) or extracellular vesicles containing proteins (e.g., platelet-derived growth factor receptor) and microRNAs that contribute to angiogenic activity [17, 41–44]. To improve the angiogenic properties (endothelial differentiation and angiogenic factor secretion), hMSCs can be genetically modified by overexpressing granulocyte chemotactic protein 2 (GCP-2), angiopoietin-1 (Ang-1), or hepatocyte growth factor to enhance their therapeutic effects on myocardial infarction [45–47]. Moreover, hMSCs may activate endogenous cardiac stem cells for myocardial regeneration through the paracrine mechanisms or direct cell-to-cell contact with cardiac stem cells [41, 48]. Combination cell therapy of hMSCs and human cardiac stem cells was more effective in reducing myocardial infarct size as compared to using either cell therapy alone [49].

On the other hand, it has been suggested that hMSCs may provide myocardial protection by suppressing inflammation within the ischemic cardiac tissue through their potent immunomodulatory capabilities [50]. hMSCs were found to promote M2 monocyte polarization (a conversion process from cardiotoxic M1 monocytes to anti-inflammatory and cardioprotective M2 monocytes) by enhancing expression of CD73 on monocytes. These CD73^+^ monocytes produce adenosine (a strong anti-inflammatory purine nucleoside) that is capable of inhibiting activation of other immune cells [51, 52]. Evidence has demonstrated that transplanted MSCs promote CD73 expression on host macrophages in the animal models of myocardial infarction [52].

Recently, cardiac adipose tissue has been identified as a novel source of MSCs. Unlike other MSCs, they possess cardiac-like and endothelial phenotypes that may greatly enhance their therapeutic potential in myocardial infarction [53]. Interestingly, it was found that transplanted cardiac adipose-derived MSCs are superior to MSCs derived from bone marrow and subcutaneous adipose tissues in the reduction of myocardial infarct size in the animal models of myocardial infarction under similar experimental conditions [54]. Like other hMSCs, cardiac adipose-derived MSCs repair infarcted myocardial tissues by promoting endogenous neovascularization through paracrine mechanisms or endothelial differentiation [53]. Evidence has shown that cardiac adipose-derived hMSCs are more effective than those isolated from subcutaneous adipose tissues in terms of inducing neovascularization [55]. Since cardiac cells are constantly subjected to electrical and mechanical signals, electromechanically stimulated cardiac adipose-derived hMSCs could be a promising therapeutic strategy for myocardial infarction [56].

**Figure 2:**
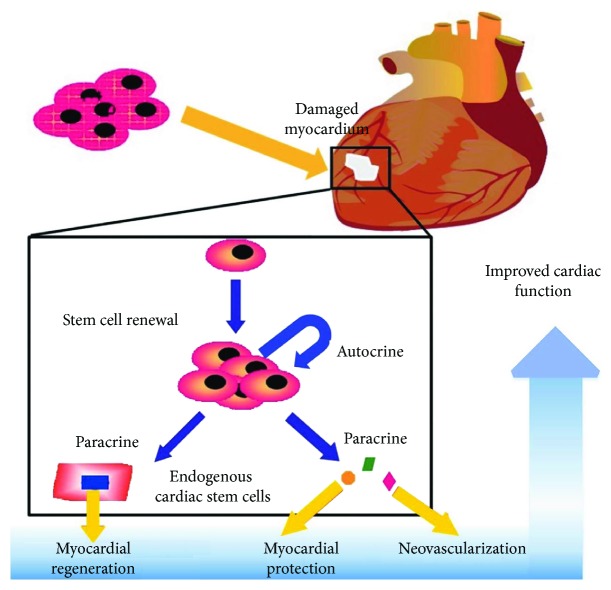
Implantation of hMSCs may repair damaged myocardium via the paracrine mechanisms. hMSCs can secrete various angiogenic factors to support neovascularization, myocardial protection, and regeneration, leading to improved cardiac function in myocardial infarction models. This image is adapted from [108] published under the Creative Common Attribution License.

Taken together, following a successful reperfusion therapy, transplantation of hMSCs may restore blood flow to the ischemic heart, reduce infarct size, and restore the left heart ventricular function through angiogenesis, myocardial protection, and regeneration (Figure 2). To date, several published clinical trials of myocardial infarction have revealed that transplanted hMSCs reduce the infarct size, improve the heart contractility, or improve the left ventricular ejection fraction with a low risk of adverse effects [57]. The clinical trial database (http://www.clinicaltrials.gov) demonstrates 33 ongoing registered clinical trials of hMSCs at phase 1 or phase 2 for myocardial infarction.

### 3.2. Ischemic Stroke

Ischemic stroke is one of the leading causes of human mortality and morbidity (e.g., permanent neurological disability) worldwide. It is resulted from occlusion of the artery supplying the blood to the brain due to embolus or thrombus, leading to loss of neural cells and disruption of brain function [21, 58]. Preclinical trials of ischemic stroke using animal models of cerebral artery occlusion have shown that implanted hMSCs may trigger angiogenesis, which in turn can enhance neurogenesis and neurological functional recovery. Implantation of hMSCs has been demonstrated to induce angiogenesis at the border zone of ischemic neural tissues through the paracrine mechanisms. In overall, hMSCs were found to secrete various angiogenic factors, including VEGF and Ang-1, to promote endogenous neovascularization [18, 59, 60]. Upon deprivation of oxygen and glucose, neurons release *γ*-secretase, a protease which upregulates the expression of key Notch-1 signaling components in hMSCs, such as Notch-1, Notch-1 intercellular domain, and Hes-1, resulting in activation of Notch-1 signaling. This signaling enhances the expression of hypoxia-inducible factor (HIF)-1*α* and increases the secretion of VEGF from hMSCs [59]. In addition, Hes-1 may inhibit the activity of phosphatase and tension homolog (PTEN) and thus further upregulating the secretion of VEGF [61]. Ang-1 released by hMSCs stabilizes the new blood vessels formed in response to VEGF by inducing vessel sprouting and branching. These vessels become resistant to leak and damage by inflammatory cells and soluble factors [60].

**Figure 3:**
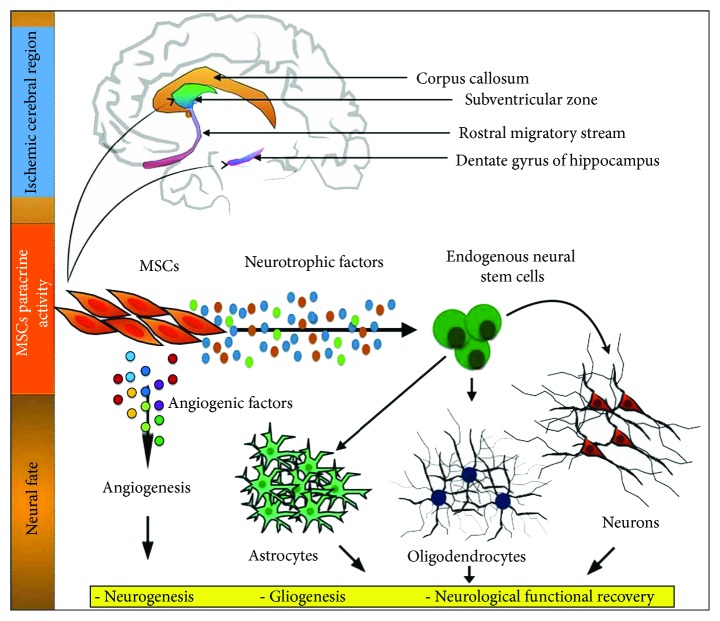
Implantation of hMSCs may repair damaged neural cells induced by stroke through the paracrine mechanisms. hMSCs can secrete various angiogenic and neurotrophic factors to support endogenous angiogenesis, neurogenesis, and gliogenesis, leading to improved neurological function in ischemic stroke models. This image is adapted from [109] published under the Creative Common Attribution License.

Besides VEGF and Ang-1, in response to HIF-1*α*, hMSCs secrete neurotrophic factors (e.g., glial-derived neurotrophic factor and brain-derived neurotrophic factor) to support survival and proliferation of endogenous neural progenitor cells and subsequently mediate their differentiation into mature neurons or glial cells [62, 63]. Moreover, hMSCs may secrete platelet-derived growth factor (PDGF) to promote M2 macrophage polarization (a conversion process from neurotoxic M1 macrophage to anti-inflammatory and neuroprotective M2 macrophage) for neurovascular and neuronal remodeling [13]. PDGF may also induce proliferation of vascular smooth muscle cells for arteriogenesis [13]. In short, hMSCs may secrete various soluble factors to promote endogenous angiogenesis for restoring blood to the brain, support endogenous neurogenesis, and restore the neurological functions (as indicated by the improved motor function, coordination, and reflex response) (Figure 3). To date, several published clinical trials of ischemic stroke have shown that transplantation of hMSCs into patients with successful reperfusion therapy reduce the stroke lesion volume and promote the neurological functional recovery. This success is indicated by improvement in the human functional behavioral and sensorimotor assessments, such as modified Rankin scale score, Barthel index, Fugl-Meyer scale, European stroke scale, and National Institutes of Health Stroke Scale [64, 65]. The clinical trial database (http://www.clinicaltrials.gov) demonstrates 15 ongoing registered clinical trials of hMSCs at phase 1 or phase 2 for ischemic stroke.

### 3.3. Critical Limb Ischemia

Critical limb ischemia is an obstruction of the arteries that reduces blood flow to the limbs, leading to permanent disability and eventually, limb loss [66]. Preclinical trials of critical limb ischemia using the animal models of hindlimb ischemia have demonstrated that hMSCs may induce angiogenesis and rescue the ischemic hindlimbs. For instance, transplanted hMSCs were found to undergo *in vivo* differentiation into endothelial cells, as indicated by the presence of human endothelial markers, such as CD31, CD34, vascular endothelial cadherin, endothelial nitric oxide synthase and vWf, and the formation of new blood vessels [67, 68]. It was shown that hindlimb ischemia models treated with hMSCs isolated from adipose tissues have a better recovery of blood flow and higher limb salvage rate than those treated with hMSCs derived from bone marrow [69]. It was found that hMSCs isolated from adipose tissues express a high level of MMP-3 and MMP-9, which may increase the release of VEGF, enabling hMSCs to efficiently differentiate into endothelial cells [69]. Further improvement of the limb salvage rate was observed in the ischemic hindlimb models treated with 3DCM composed of hMSCs on a substrate with immobilized bFGF [68]. Hypoxic conditioning and genetic modification of hMSCs can further increase the secretion of angiogenic factors, such as VEGF, bFGF, PDGF, and Ang-1, resulting in an enhanced angiogenesis and recovery of blood flow to the ischemic hindlimbs [19, 70–72]. Collectively, hMSCs may restore blood flow to the ischemic hindlimb and thus, prevent limb loss via endothelial differentiation and paracrine mechanisms. To date, several published clinical trials of critical limb ischemia have shown that transplantation of hMSCs into patients with successful reperfusion therapy reduce leg ulcer size, restore limb perfusion (as indicated by improvement in ankle-brachial index and transcutaneous oxygen measurement at the limbs), or recover limb function (as indicated by improvement in pain-free walking distance and time) [22, 73]. The clinical trial database (http://www.clinicaltrials.gov) demonstrates 10 ongoing registered clinical trials of hMSCs at phase 1 or phase 2 for critical limb ischemia.

## 4. Challenges Associated with Future Translation of hMSCs to Ischemic Disease Therapy

There are some advantages and limitations of using hMSCs for the treatment of ischemic tissues (Table 1). Overall, many positive outcomes have been reported in preclinical trials utilizing hMSCs to treat ischemia-related medical conditions. These results have spurred ongoing clinical trials. However, the benefits observed in preclinical animal models have not yet been reproduced consistently in humans using conventional cell therapies. Conventional cell infusion approaches have been associated with complications, such as poor cell engraftment, short duration of cell persistence in the host, and low cell survival rate, in both animal models and human [74, 75]. These are likely due in part to the loss of homing and engraftment potential in cultured hMSCs after expanding under conventional cell culture conditions [74, 76]. Additionally, some animal models may not accurately mimic the conditions of human diseases [20]. Taken together, these may lower the successful rate of translation from animal models to clinical trials. Hence, there are increasing number of studies focused on techniques involving bioprocessing and tissue engineering (e.g., *in vitro* human tissue models, scaffold, and cellular imaging) to improve the translation rate of hMSCs. For example, a bioactive construct can be created by combining hMSCs with natural or synthetic scaffolds that possess similar characteristics as the native tissues to improve its integration into target ischemic tissues for tissue repair and regeneration. Moreover, such bioactive construct can be used on human *in vitro* ischemic disease models to evaluate its effectiveness prior to clinical trials.

### 4.1. Bioprocessing of hMSCs

There are a number of challenges that need to be addressed before hMSCs can achieve their widespread clinical applications. For example, in studies published to date, the MSC cell populations used were isolated from a number of varieties of tissues using different methodologies and expanded in culture using nonstandard protocols. In addition, many of the reported studies utilized cells exposed to animal-based serum. Standard culturing methods need to be developed, tested, and implemented to adhere to regulatory standards, achieve safe and reproducible results in a clinical setting, and make them translational. Important bioprocessing considerations include identification of proper cell source (autologous versus allogeneic), cell locations (e.g., bone marrow, adipose tissue, and synovial membrane), culture medium, static versus dynamic (bioreactor-based) culture, and long-term storage. Moreover, other considerations related to the administration of cells include optimization of cell dosage, cell administration method (cell alone versus cell-laden scaffold), and timing. Although work to address some of these important concerns is already well underway [15, 16, 77–81], it would take a considerable amount of time before these cells can be widely implemented clinically to treat ischemic tissues.

To date, significant efforts have been devoted towards the translation of hMSC-based therapy such as using bioreactors for cell population expansion and differentiation, developing animal component-free growth media, and generating cell banking protocols [82–84]. First, it is essential to optimize the procedures for isolation, expansion, and storage of hMSCs to ensure that only hMSCs are expanded within a specific duration for avoiding long-term expansion, which would increase the risk of harmful genetic transformation. For instance, a microcarrier-based stirred suspension bioreactor has demonstrated its capability to expand hMSCs from 5 × 10^5^ cells to a clinically relevant cell number (6 × 10^8^ cells) within a short duration (33 days) [16]. Besides that, hMSCs can be stored in master cell banks and comprehensively characterized at the time prior to clinical use. The large-scale production of safe and effective hMSCs should be in compliance with current good manufacturing practice (GMP) regulations. With the use of bioreactors incorporated with in-process control, this may automate the workflow and monitor the culture conditions, resulting in a good yield of clinical-grade cells. Additionally, the growth medium of hMSCs must be free of animal products, and the media formulations must be fully well-defined [15, 80]. For administration of hMSCs treatment in patients, a strictly accessible environment is required to reduce batch-to-batch variations of hMSCs and reduce the risk of contamination.

### 4.2. Development of a Functional Human *In Vitro* Ischemic Disease Model

One notable challenge has been the lack of a functional human *in vitro* ischemic disease model in which research can be easily undertaken to determine the details and efficacy of hMSC implementation. The only viable option to date has been the use of animals, which has ethical concerns and is costly and only available as an option to those who have the proper animal care facilities. Development of human *in vitro* ischemic disease tissue models is certainly a crucial need to overcome this research bottleneck. Three-dimensional biomimetic *in vitro* tissue models are conducive to the investigation of tissue or cell physiology in a systematic and repetitive manner, less time consuming, less expensive, allow high-throughput testing, and can be used in a standard manner across different research facilities [85]. This could facilitate the rapid translation of promising lab results to the clinic.

With the advances in microfluidic and hydrogel fabrication technologies, multiple techniques (e.g., needle-based molding, dissolvable network-based sacrificial molding, and bioprinting) have tremendous potential to generate 3D biomimetic blood vessels that can be used to develop an *in vitro* ischemic disease and atherosclerotic models [86]. Among the microfabrication techniques, needle-based molding has been mostly utilized to develop biomimetic blood vessels for many applications, e.g., drug permeability testing and mechanical studies [87, 88]. A cylindrical vessel is generated by preinserting a cylindrical object (e.g., wire, needle, or rod) followed by casting a hydrogel (e.g., collagen type I and fibrin) around it and finally removing the cylindrical object after gel polymerization. This simple method is constrained to the fabrication of simple linear cylindrical vessels [89]. This vessel can, however, be functionalized with human endothelial cells cultured at the inner wall of the vessel while human perivascular cells (e.g., vascular smooth muscle cells, pericytes, and astrocytes) cultured in the wall surrounding the vessel [89, 90]. Following functionalization, this blood vessel can be subjected to biochemical cues (e.g., low-density lipoprotein, high-density lipoprotein, and human whole blood) and mechanical cues (e.g., flow-induced shear stress) to create functional human *in vitro* atherosclerotic models [91, 92]. This biomimetic model would be useful for exploring potential therapeutic effects of hMSCs in atherosclerosis for the prevention of tissue ischemia. If hMSCs can successfully treat atherosclerosis and repair injured blood vessels, hMSC transplant may be able to replace reperfusion therapy. Moreover, with the addition of organ-specific cells (e.g., cardiomyocytes and neurons) and blood components into these atherosclerotic models, *in vitro* functional human ischemic heart or brain disease tissue models can be developed. Such biomimetic *in vitro* disease models may assist in comprehensively elucidating the therapeutic mechanisms of hMSCs in the ischemic diseases, which allows for optimization of their cell processing procedures and improvement of their therapeutic benefits for ischemic diseases.

For dissolvable network-based sacrificial molding technique, a preformed cylindrical vessel made by sacrificial material (e.g., gelatin or Pluronic F127 fugitive ink) is first encapsulated in a hydrogel. Gelatin or Pluronic F127 fugitive ink is eventually liquefied by incubation at 37°C or 4°C under a modest vacuum, respectively, and removed from the hydrogel after gel polymerization to form a hollow cylindrical vessel in the hydrogel [93, 94]. In recent, bioprinting integrated with the dissolvable network-based sacrificial molding and layer-by-layer bonding technique is developed to print living cells in a 3D structure with a vessel. However, some limitations (e.g., repeatability and resolution for 3D vascular structure construction, required cell density, and cell damage due to high-speed deposition) still remain unresolved [86]. These methods can also be used to generate a functional human *in vitro* ischemic disease model for hMSC therapy.

### 4.3. Engraftment and Persistence of Transplanted hMSCs in the Host

Engraftment and persistence of transplanted hMSCs in a host are major challenges to be solved in stem-cell based therapy [76]. It seems that the engraftment rate of hMSCs is low, and hMSCs may not persist for longer periods in a host, thereby may reduce their therapeutic efficacy. For instance, whereas a majority of hMSCs were found to be dead within 3 days after implantation in the animal models of myocardial infarction, positive outcomes were still observed [75, 95]. Treatment of hMSCs on a functional human ischemic disease model can enable researchers to determine hMSC engraftment rates, and evaluate the persistence of these cells within tissues by using a noninvasive cellular imaging modality. Imaging modalities, such as magnetic resonance imaging and bioluminescence imaging, have been shown to be capable of tracking transplanted stem cells in animal models of ischemic stroke and myocardial infarction, respectively, in real-time [96, 97]. This may help to explore the underlying mechanisms of stem cell therapy for ischemic diseases. Moreover, both engraftment and persistence of hMSCs can be improved with various methods (e.g., cytokines, hypoxia, and material-based approach) which may potentially improve their therapeutic efficacy [95, 98, 99]. For instance, 3D fibrin-cell patches were found to improve persistence duration and survival of transplanted hMSCs in the animal models of myocardial infarction, thereby improved the cardiac functions [100]. This scaffold may protect hMSCs from anoikis and improve interaction between hMSCs and ischemic tissues for effective stem cell therapies. By having the human *in vitro* ischemic disease models, these modified hMSCs are readily to be tested *in vitro*, which is something that is difficult to be performed in a clinical trial.

## 5. Conclusions

hMSCs have demonstrated their potential therapeutic effects on the ischemic diseases (including myocardial infarction, stroke, and critical limb ischemia) due to their excellent properties in immunomodulation, angiogenesis, and paracrine secretion of bioactive factors. The mode of action of hMSCs on the ischemic diseases is by means of paracrine mechanisms, endothelial differentiation, or direct cell-to-cell contact. However, their comprehensive therapeutic mechanisms in ischemic diseases, especially in attenuating atherosclerosis (main culprit for the ischemic diseases), remain elusive which requires further investigation. With the advances in microfluidic and hydrogel fabrication technologies, human *in vitro* ischemic disease tissue models are expected to be developed rapidly to accelerate the development of efficient hMSC-based therapeutics. With the exceptional and fascinating properties of hMSCs, it is envisioned that hMSCs would be an excellent cell source for a wide variety of clinical applications, including but not limited to ischemic diseases.

## Figures and Tables

**Table 1:** The advantages and limitations of hMSCs for the treatment of ischemic tissues.

Advantages	Limitations
hMSCs may repair injured vessels and ischemic tissues through their unique immunomodulation properties and paracrine mechanisms [13].	It is difficult to obtain sufficient numbers of healthy autologous hMSCs from elderly patients or patients with severe diseases [101].
hMSCs can be isolated from various locations within the human body and easily expanded *in vitro* [102].	The successful rate of differentiation of transplanted hMSCs into fully functional cardiomyocytes or neurons in a recipient remains elusive [103].
hMSCs provide a less invasive treatment procedure with low risk of adverse effects compared to surgical and pharmacological approaches [104].	hMSCs have a limited replicative lifespan [107].
hMSCs are relatively well characterized, and its clinical use can avoid the ethical concerns related to embryonic stem cells [105, 106].	
